# Stereochemical Terminology in Chiral Drugs: Still Between Confusion and Misinterpretation

**DOI:** 10.1021/acsmedchemlett.6c00195

**Published:** 2026-04-28

**Authors:** Juan García de la Concepción, Anthony J. Burke, Pedro Cintas

**Affiliations:** † Departamento de Química Orgánica e Inorgánica, Facultad de Ciencias, and Instituto del Agua, Cambio Climático y Sostenibilidad (IACYS), 16759 Universidad de Extremadura, 06006 Badajoz, Spain; ‡ Coimbra Chemistry Centre−Institute of Molecular Sciences (CQC−IMS), Departamento de Química, 37829 University of Coimbra, 3004-535 Coimbra, Portugal; § Pharmaceutical Chemistry Laboratory, Faculty of Pharmacy, Pólo das Ciências da Saúde, Azinhaga de Santa Comba, University of Coimbra, 3000-548 Coimbra, Portugal; ∥ Center for Neurosciences and Cellular Biology (CNC), Polo I, Rua Larga, Faculdade de Medicina, University of Coimbra, 3004-504 Coimbra, Portugal

**Keywords:** chiral drug, molecular handedness, configurational notation, chiroptical rotation, academic training

## Abstract

The Viewpoint addresses the importance of correct designations of stereochemistry in chiral drugs. Sources of confusion typically arise from merging disparate concepts such as handedness, configuration, and chiroptical properties. This Viewpoint recommends the consistent use of configurational labeling, while avoiding ambiguous and context-dependent nomenclature.

## Graphical abstract

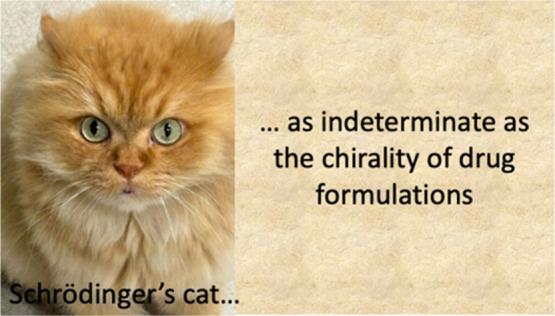

Chirality and stereorecognition are intimately intertwined. Most receptors and biomolecules are inherently chiral (actually enantiomerically pure entities), and numerous chiral drugs are common APIs. As once declared by Francis Crick, even preceding his central dogma of molecular biology, “*the first great unifying principle of biochemistry is that the key molecules have the same hand in all organisms*”.^1^ This aspect is well portrayed by the homochirality shown by naturally occurring L-amino acids or D-sugars, as well as the helical arrangement of nucleic acids. Clearly, it should be unnecessary to emphasize the importance of chirality, as some monographs and textbooks in medicinal chemistry highlights the relevance of stereochemistry and topology in drug action.^2^ Still, it is a difficult matter for students and senior scientists alike, who may also be unfamiliar with organic stereochemistry and its vocabulary.^3^ A case is made in this Viewpoint for increasing the clarity in medicinal chemistry language involving chiral, nonracemic, or enantiopure drugs and formulations. Moreover, precision appears to be compulsory given the ascending trend to bring single enantiomers to the pharmaceutical market as revealed by new approvals in the past 15 years.^4^ In contrast, there has been a concomitant decrease in FDA-approved small molecule drugs as racemates, although the development of a racemic drug can be relevant in terms of cost and safety against endemic infectious diseases.^5^

While there is a well-defined and established IUPAC terminology for chiral molecules, it is either ignored or applied incorrectly in numerous publications dealing with chiral drugs. To a significant extent, a confusion in literature arises from the misuse of stereodescriptors and/or the semantic corruption of others. A classic example that often mislead students is provided by L-dopa (**1**) or levodopa (also hyphenated and italicized, *levo*-dopa, which adds extra confusion). Both terms denote obviously the same molecule, but the prefixes are not related to each other as “L” indicates the same configuration as the parent amino acid derivative. L-Dopa can be formulated as either L-3,4-dihydroxyphenylalanine or L-3-hydroxytyrosine. Like most proteinogenic amino acids (with the sole exception of L-cysteine), L-dopa has the absolute (*S*)-configuration (Cahn–Ingold–Prelog system). In contrast, *levo* (from Latin *laevo*) merely denotes the levorotatory enantiomer (i.e., the plane of polarized light is deflected to the left) at a given wavelength (usually the sodium D-line at λ = 589 nm). Although measuring chiroptical profiles of enantiomers or chiral materials (e.g., ellipticity or Cotton effects) is useful for configurational assignments, the optical rotation *per se* lacks configurational information. Note that one enantiomer can eventually exhibit opposite signs at different wavelengths and both the magnitude and sign may be dependent on the solvent as well.
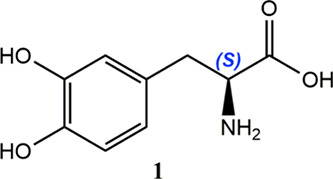


It is well-known that numerous homochiral (i.e., enantiomerically pure) drugs are dextro- or levo-rotatory irrespective of their absolute configurations, either (*R*) or (*S*) so long as one chiral center is present. Archetypal eutomers (i.e., the more potent enantiomer) include (*R*)-(−)-methadone (opioid activity), (*S*)-(−)-warfarin (anticoagulant), or (*R*)-(−)-salbutamol (bronchodilating effect), the latter introduced as levalbuterol (*vide infra*).^6^ Likewise, in nonsteroidal anti-inflammatory aryl-substituted propanoic acids (profens), the eutomer activity generally resides on the (*S*)-configured enantiomer having a greater inhibition of COX isoforms than the (*R*)-distomer (the less potent isomer), which applies to blockbuster drugs such as (*S*)-(+)-ibuprofen and (*S*)-(+)-naproxen (**2**). For such a family, the terms D or L make no sense, as the configuration of profens cannot be related to the structural handedness of D-sugars or L-amino acids. Neither do they have a direct correlation with *R/S*-configurations. Historically, D-labeled configuration arises from the structural similarity, i.e. right-handed assignment, to D-glyceraldehyde, the simplest chiral carbohydrate, which has (*R*)-configuration and is dextrorotatory (it rotates the plane of polarized light to the right). Again, the sign of optical rotation is purely casual. Confusingly, the name D-(+)-naproxen has been reported in recent literature,^7^ thereby suggesting an intentional correlation with a dextrorotatory isomer in the D-sugar series. The term L-naproxen can also be found in both Web sites and literature sources to purportedly denote the left-handed enantiomer.

Anyhow, the pervasive association of molecular handedness (i.e., configuration) and optical rotation represents an overlooked element that often escapes peer review as well. In a recent and otherwise interesting article that appeared in a high-profile journal, we are told that “most naturally occurring proteins are levorotatory (L), made up of only L-amino acids.... Amino acids in nature are predominantly left-handed or levorotatory (L), whereas their enantiomers are right-handed or dextrorotatory (D)”.^8^ After a stimulating feedback between one of us and the corresponding author, they ultimately grasped that handedness and optical rotation have nothing to do (obviously with opposite signs for two incongruent mirror-image biomolecules). In context, the prefixes *d*- and *l*-, always typed in lower case and italicized, should be employed to denote dextro- and levo-rotatory enantiomers, and not their configurations. Unfortunately, this meaning cannot be unambiguously established in practical terms across the scientific literature.

In context, the terms right-handed and left-handed, while useful for comparative purposes such as chiral macromolecules of opposite chirality, are unfortunate to design the configuration of small molecules. Such terms indicate the sense of twist of a propeller or helix; it is right handed (denoted conventionally with symbol *P* for plus) if the twist is clockwise when one progresses along the axis away from the observer, and left handed (symbol *M* for minus) if the twist is counterclockwise. However, and once again, they do not correlate with the absolute directions of chiroptical properties (electric and magnetic transition moments).^9^ To complicate things further, the *helix* nomenclature and the *chiral axis* nomenclature employed for atropisomers (see below) are not coincidental either.

With the advent and progressive introduction of *chiral switches*, namely medications approved or marketed as racemates and subsequently redeveloped into more potent single enantiomers,^10,11^ distinctive names have been applied to the homochiral drugs. Typical examples include esomeprazole, levofloxacin, esketamine, levoleucovorin, escitalopram, dexketoprofen, or dexmetomidine, among others, for which the mixed vocabulary of handedness and chiroptical rotation coexist. Thus, dexketoprofen, (*S*)-(+)-ketoprofen (**3**), corresponds to the chiral switch of *rac*-ketoprofen, which could be better designated as esketoprofen (not coined, nevertheless).
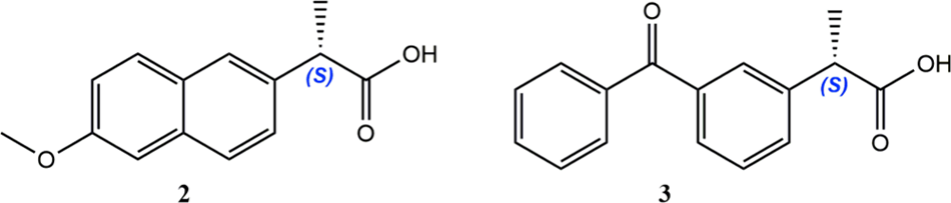


Likewise, levobupivacaine (by chance the levorotatory isomer) is actually the (*S*)-configured enantiomer having a longer anesthetic action than the racemate. The antihistaminic agent dexchlorpheniramine (Polaramine) represents the (*S*)-(+)-enantiomer, which is about 200 times more potent than the (*R*)-(−)-distomer.^2^ Clearly, the terms esbupivacaine and eschlorpheniramine would be much more mnemonic.

When multiple stereogenic elements are involved, the correct assignment of their configurations provides an unambiguous specification, although this detail can be tedious. Shortcuts to shorter names or descriptors may be valid if structural fragments of known chirality can be identified. An illustrative case is (*R*)-baicalin (**4**), a flavone glycoside that induces apoptosis and hence potentially inhibits tumor growth. In a recent study, the homochiral dual combination of this substance with a complex saponin, astragaloside IV (**5**), whose enantiomers can likewise be designed *R* or *S*, holds promise for treating epithelial ovarian cancer.^12^ Such configurational assignments are far from being obvious, albeit they might be acceptable if one bears in mind that these APIs are glycosides derived from all-D-configured carbohydrates (Figure 1). While the phenolic aglycone of **4** is achiral, the chiral fragment derives from a β-D-glucopyranosic acid. In astragaloside IV (**5**), the aglycone is a chiral pentacyclic terpenoid, but the molecule as a whole is likewise a double glycoside containing β-D-xylopyranosyl and β-D-glucopyranosyl residues. For D-configured hexopyranosides, the stereochemistry at C5 is invariably *R* and, conversely it will be *S*-configured in the L-series. Accordingly, (*R*)-baicalin and (*R*)-astragaloside IV only contain D-configured sugar residues.^12^ The corresponding (*S*)-enantiomers are instead composed of the rare L-configured sugars.^13^ In the present case at least, the names D-baicalin or D-astragaloside IV would be appropriate as well. Certainly, the latter represents a limiting case where further stereocenter location would be desirable, albeit the configurational notation of sugar skeleta is unequivocal.

**Figure 1:**
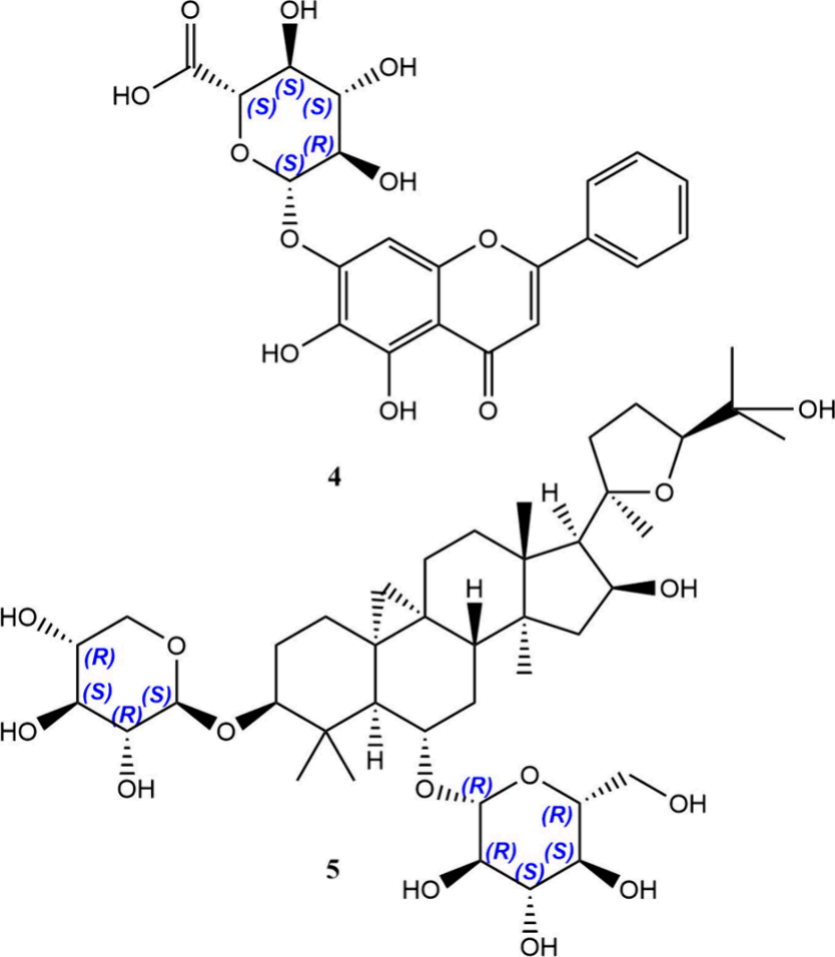
Natural isomers of baicalin (**4**) and astragaloside IV (**5**), both containing D-configured monosaccharides. For clarity, only stereodescriptors at the sugar moieties are shown.

An intermediate case is given by vicinal stereocenters, another structural motif present in various drugs, with methylphenidate (sold as the corresponding hydrochloride) as paradigmatic example. The drug has been a useful treatment against attention-deficit/hyperactivity disorders (ADHD). The two chiral carbons afford four stereoisomers that comprise two diastereomers, each being a racemate of enantiomorphous molecules. From the onset, the methyphenidate diastereomers were named *erythro*- and *threo*-methylphenidate, thus mimicking the structural arrangements shown by the consecutive chiral atoms of sugar tetroses, erythrose and threose. Unfortunately, such a nomenclature has proven to be problematic (awful if one may say so). First, *erythro* and *threo* descriptors denote relative configurations and are therefore conformation-dependent. Viewed in staggered conformation, D- and L-*threo* enantiomers show the substituents at the stereocenters in *syn* disposition, while they display an antiperiplanar orientation in D- and L-*erythro* isomers. On the other hand, the racemic *threo* pair (**6a**/**6b**) exhibits the highest potency against ADHD and gave rise to the first chiral switch of the drug, namely ritalin. At this stage, it is convenient to state that the absolute configurations of ritalin enantiomers, (*R,R*) and (*S,S*), mismatch those of D- and L-threose, (*S,R*) and (*R,S*), respectively, thus lacking the appropriate stereochemical correlation. The most potent dextrorotatory isomer (**6a**), having (*R,R*) vicinal configurations, has resulted in the second chiral switch, focalin, released as dexmethylphenidate, which has not removed *rac*-ritalin from the ADHD market, nevertheless. The double chiral-switch strategy has been detailed recently by Agranat and D’Acquarica, who in turn recommend to abandon the sugar-based stereodescriptors *erythro* and *threo*.^14^ In view of our above premises, we fully agree. Vicinal stereocenters in tramadol, the worldwide used μ-opioid receptor analgesic, engender the same confusion as the two diastereomers are denoted *cis* and *trans* (i.e., the relative orientations of the highest priority substituents), with the *cis*-racemate comprising the mixture of (*R,R*) and (*S,S*)-enantiomers (**7a**/**7b**) being the prescribed drug.^15^ 
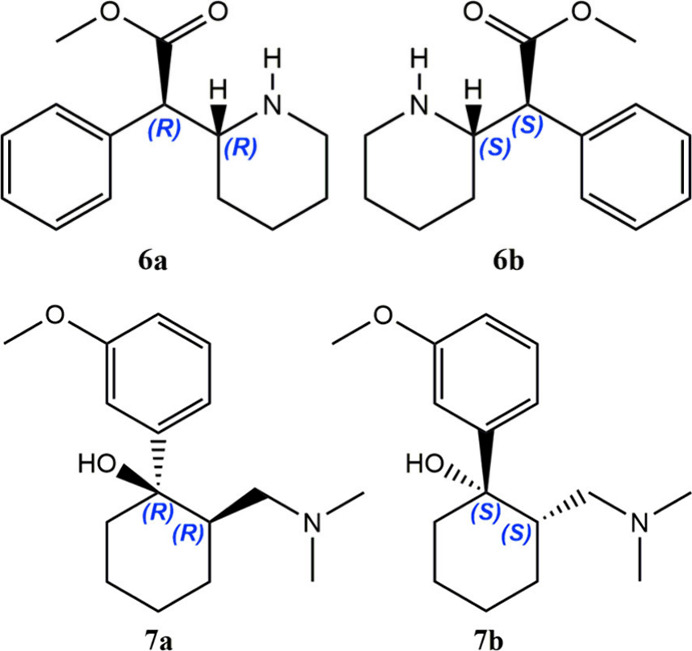


Drugmakers have known the potentiality of atropisomers for at least two decades,^16^ albeit the field is in the infancy relative to drugs based on stereocenter chirality. The major challenge involves the design of configurationally stable rotational isomers, which generally requires energy barriers above ∼24 kcal/mol.^17^ A salient example is provided by the first-in-class KRAS G12C inhibitor sotorasib targeting nonsmall cell lung cancer, which has been approved in both the US and the European Union. From a structural standpoint, sotorasib is especially relevant as it contains a (*S*)-configured atom at the methyl-substituted piperazine ring plus two chiral axis, although only the *o,o*′-dialkyl-substituted pyridine hinders enough rotation (with a barrier >40 kcal/mol) for atropisomerism to occur at room temperature. Eutomer activity resides in the (*M*)-stereoisomer (**8**) using the helicity notation, as typically shown. Since the correspondence of *aR* with *M* and *aS* with *P* is general,^9^ we contend that axial chirality descriptors constitute the best way to unequivocally assign the configuration of atropisomeric drugs; here (*aR*,*S*)-sotorasib for the desired enantiomer. On the other hand, it is expected that installation of axial chirality will be a postsynthetic protocol to ensure racemization-free design, as exemplified by the construction of an advanced sotorasib intermediate with recycling of the undesired distomer.^18^ 
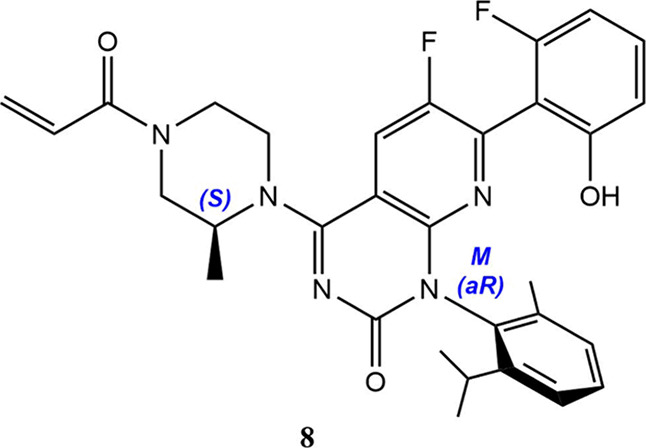


To sum up, it is surprising that despite having been mentioned repeatedly the confusion involving the various forms of naming chiral drugs, attempts to establish a coherent terminology remain unsolved. In this Viewpoint, we have highlighted a few representative cases where flawed notations and/or stereochemically ambiguous descriptors result in vague characterization of the active ingredients of chiral drugs. We would like to recommend a series of tips, which might be suitable for new chiral entities under therapeutic evaluation.

- Avoid the use of *dex*, *dextro*, *levo*, and related prefixes, which simply denote the sign of optical activity. They are devoid of structural information and, above all, do not correlate with molecular handedness. Stereodescriptors *d* and *l* are equivalent to dextrorotatory and levorotatory, respectively, and are to be discouraged as well. Although the literature is plagued by such prefixes, often mixed with other descriptors, it is unclear if they refer to optical rotation or configuration.
- Although chirality and handedness can be interchanged as synonyms, avoid the thoughtless use of *right-handed* and *left-handed* to denote the configuration of APIs, as in numerous cases they convey the erroneous association with the sign of rotation.
- The use of D- and L-stereodescriptors should largely be restricted to drugs or fragments thereof that can be correlated with parent amino acids or sugars in a straightforward manner. Drugs based on peptides and monosaccharides involving multiple stereocenters benefit from such configurational relationships.
- For the large set of drugs containing one or two stereocenters, the conventional *R/S* notation represents the best way of naming in line with the CIP system. The use of prefixes *es*- (for *S*) and *ar*- (for *R*) accompanying the name, should be encouraged, as reflected by the unambiguous structural notation already employed in some chiral drugs.
- It is noteworthy that for chiral drugs containing chiral axis, *atropisomers*, their helicity specified through the stereodescriptors *P* (clockwise) and *M* (counterclockwise) can be made equivalent to *aS* and *aR*, respectively. Accordingly, for naming atropisomeric drugs, the prefixes *es*- and *ar*- should be used instead of *P* and *M*, respectively.

Lastly, focusing on the inconsistency in nomenclature is beyond simple terminological formalities. Misuse and ignorance leads to ambiguity and misconceptions on the nature and properties of stereoisomers. Moreover, accurate designations enable proper conclusions on the relationships between chirality and biological activity.

## Notes

Neither AI tools nor generative language have been employed in the preparation of this manuscript.
